# Solitary kidney and thirteen (13) tumors: Laparoscopic radical nephroureterectomy, bench ex-vivo nephron-sparing surgery and auto-transplantation

**DOI:** 10.1590/S1677-5538.IBJU.2019.0443

**Published:** 2020-09-02

**Authors:** Luis Alberto Zanettini, Luis Felipe Snel Zanettini, Alan Paulmichl, Antonio Carlos Zanettini

**Affiliations:** 1 Serviço de Urologia Hospital Unimed Nordeste Caxias do SulRS Brasil Serviço de Urologia, Hospital Unimed Nordeste, Caxias do Sul, RS, Brasil;; 2 Universidade Federal do Espírito Santo Centro de Ciências da Saúde Vitória Brasil Universidade Federal do Espírito Santo, Centro de Ciências da Saúde, Vitória, Brasil;; 3 Serviço de Urologia Unimed Nordeste Caxias do SulRS Brasil Serviço de Urologia, Unimed Nordeste, Caxias do Sul, RS, Brasil

## INTRODUCTION

Renal autotransplantation is a rare, safe and effective surgical procedure for the treatment of complex urological conditions and used in selected patients ( 1 ).

For single kidney tumors, multiple bilateral tumors and in impaired renal function patient tumors, it is mandatory to preserve as many nephrons as possible. This goal can be achieved either by the laparoscopy or open approaches. In a small proportion of patients with complex tumors, in situ tumor resection is not feasible. In such situation, ex-vivo tumor resection and autotransplantation are advocated. The main reason for perform a kidney autotransplantation is to preserve renal parenchyma ( 1 - 4 ).

Patients with organ-confined kidney tumors, nephron-sparing surgery improves life expectancy compared to radical nephrectomy, due to a reduced rate of non-cancer associated mortality while being oncologically safe ( 5 ).

### Case Presentation

A 44-year-old man with several tumors in a left solitary kidney was referred to our team. He was healthy, with no weight loss. Laboratory tests showed serum creatinine 1.30mg/dl, elevated serum C-reactive protein, and erythrocyte sedimentation. Reported one sister and tree uncles operated with renal tumor, two of them with bilateral tumors.

He had a right open radical nephrectomy four months before for a big Kidney tumor. Pathology reported a pT2bN0Mx multifocal Papillary Renal Carcinoma with 20.2 x 11.6cm and invasion of the capsule and collector system.

**Figure 1:**
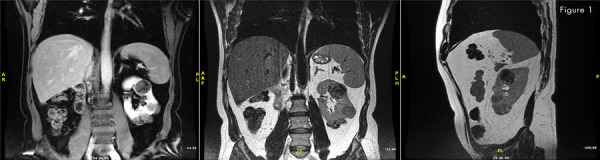
MRI report - solitary left kidney - at least eleven renal tumors.

Ultrasound (US), computed tomography (CT) and MRI showed in the solitary left kidney at least eleven renal tumors, the largest entirely exophytic with 4.2cm at the upper pole and 4.7cm and 3.7cm at the lower pole. No central tumors or involvement of the renal sinus, renal vein or cava vein. No lymphadenomegaly or metastases in abdomen or thorax ( Figure-1 ).

We discussed with the patient the options: a definitive laparoscopic radical nephrectomy, hemodialysis and kidney graft several years later or a planned conservative surgical procedure. He decided for the conservative approach.

**Figure 2:**
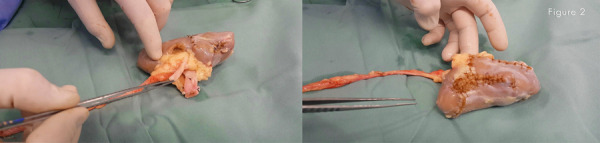
Kidney reconstructed ready to autotransplantation

A planned left transperitoneal laparoscopic radical nephroureterectomy was performed. After complete release of the kidney, the artery and the vein were proximal doubled clipped by polymer clips and cut off. The ureter was cut at crossing iliac vessels level. The kidney was withdrawn with an iliac fossa incision, was kept in ice slush and perfused with a continuous cold storage transplant solution. With ultrasonography aid, a bench nephron-sparing surgery was performed and all tumors resected or enucleated to maximally preserve the normal parenchyma. The incised calyxes were repaired and a meticulous reconstruction of the kidney with absorbable sutures and hemostatic booster interposition made. It was spent 105 minutes to resect and to reconstruct ( Figure-2 ). During the bench surgery the patient was repositioned in the dorsal decubitus and a hemodialysis catheter was inserted in the subclavian right vein. With a Gibson right incision an autotransplantation was performed. Reconstructed kidney was autotransplanted in the retroperitoneum and the vascular axis was anastomosed in the external iliac vein and artery. The ureter was implanted in the bladder by an extravesical Lich-Gregoir antireflux procedure with a double J stent. A Penrose drain was left in place.

Hemodialysis was necessary daily in the first week. On the 9^th^ post-op a great urinoma collection forced a reoperation and a suction silicon drain was left. The patient was discharged on the 19^th^ post-op with a high debit urinary fistula. He was in good physical and health condition. The fistula closed spontaneously on the 40^th^ post-op and the double J stent was withdrawn.

**Figure 3:**
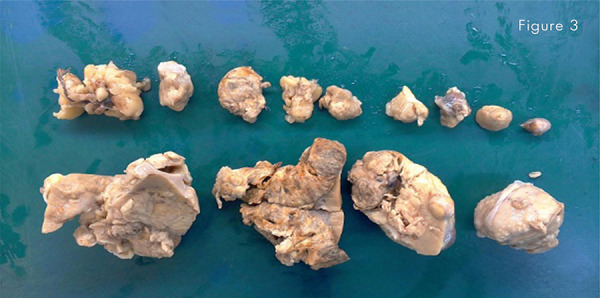
Gross appearence of papilary renal cell carcinoma: Thirteen (13) tumors ranging from 1.10cm to 6.10cm on the major axes.

Thirteen portions of the kidney were resected or enucleated. The anathomopathological report a multifocal Papillary Renal Carcinoma types 1 and 2: Cromophilus ( Figure-3 ).

The serum creatinine was monitored and dropped down progressively. It was 2.76mg/dl in the 30^th^ post-op and 1.74mg/dl in the 60^th^ post-op. No other complications occurred. No relapse tumors in abdomen MRI made on the 4^th^, 8^th^ and 12^th^ months post-op were observed. On the 13^th^ month post-op he has good health, takes a normal life, and the creatinine is 1.69mg/dl.

## DISCUSSION

Kidney autotransplantation was first performed by Hardy in 1963 for high ureteral injury ( 6 ).

After this landmark surgery, autotransplantation has been described for renal artery disease, complex urological reconstruction, upper ureteral tumors, extensive renal parenchymal tumor ( 1 , 3 ).

The term bench surgery is used to describe reconstructive surgery on diseased kidneys receiving crystalloid perfusion outside the body.

No large series is published in literature about solitary kidneys and autotransplantation surgery and limited outcomes were reported.

Patients with tumor-bearing solitary kidneys have great benefit from organ-sparing approaches avoiding the need of long-term hemodialysis. Studies reported by Dialysis Outcomes and Practice Patterns Study consistently show a marked difference in crude mortality between different Countries. In 2003, DOPPS reported the crude 1-yr mortality rates were 6.6% in Japan, 15.6% in Europe, and 21.7% in the United States ( 7 ).

In the oncological setting, recurrence rate and few complications were reported in this technique in patients with renal or ureteral tumors. In three cases of bilateral tumors and three with solitary kidney treated with retroperitoneal laparoscopic nephrectomy, bench surgery and autotransplantation only one patient died with multiple metastasis after 18 months ( 8 ).

One of the largest series of laparoscopic nephrectomies for autotransplantation was reported by Tran et al. in 2015 including 52 patients with more than 90% success rate over a 6-year follow-up length of time ( 9 ). Recurrence may occur, even after negative surgical margins at ex vivo tumor excision. It occurred in 4 (50%) of eight cases in complex centrally located renal tumors: 3 renal cell carcinoma (RCC) and 1 urothelial (CIS) renal tumor. The single death in this group was the patient with urothelial CIS, who died after tumor progression 2 years after the autotransplantation ( 9 ).

In 12 patients with complexes renal tumors undergoing radical nephrectomy and autotransplantation (five cases of renal cell carcinoma - RCC, five cases of upper urinary tract carcinoma - UTUC, one case of renal metastasis, one nephroblastoma), it was observed the following oncological outcome: recurrence or metastasis, five cases; tumor-related death, two cases; RCC progression, one case; RCC or UTUC local recurrence four cases. All patients who died had functioning kidneys ( 10 ).

Laparoscopic renal nephrectomy for autotransplantation provides an opportunity to decrease morbidity. Fabrizio et al. performed the first laparoscopic nephrectomy with open autotransplantation ( 11 ).

In conclusion, ex-vivo nephron-sparing surgery for renal tumor is a feasible option in especial situations. Laparoscopic approach should be used whenever it is possible to reduce the morbidity of the procedure. Bench surgery and autotransplant offer several advantages over the anephric condition with renal replacement therapy and it should be considered after discussing with the patient.
